# Clinical Frailty Scale score is a predictor of short-, mid- and long-term mortality in critically ill older adults (≥ 70 years) admitted to the emergency department: an observational study

**DOI:** 10.1186/s12877-024-05463-7

**Published:** 2024-10-21

**Authors:** Dariush Javadzadeh, Björn W Karlson, Joakim Alfredsson, Elin Ekerstad, Jenny Hellberg, Johan Herlitz, Niklas Ekerstad

**Affiliations:** 1 Department of Health, Medicine, and Caring Sciences, Unit of Health Care Analysis, Linköping University, Linköping, Sweden; 2 Department of Research and Development, NU Hospital Group, Trollhättan, Sweden; 3 Department of Molecular and Clinical Medicine, Institute of Medicine, Sahlgrenska Academy, University of Gothenburg, and AstraZeneca Gothenburg, GothenburgMölndal, Sweden; 4 Department of Health, Medicine and Caring Sciences and Department of Cardiology, Unit of Cardiovascular Sciences, Linköping University, Linköping, Sweden; 5 Department of Cardiology, NU Hospital Group, Trollhättan, Sweden; 6 Center for Prehospital Research, Faculty of Caring Science, Work Life and Social Welfare, University of Borås, Borås, Sweden

**Keywords:** Older adults, Emergency department, Predictors, Clinical frailty scale, Mortality

## Abstract

**Background:**

The estimated prognos of a patient might influence the expected benefit/risk ratio of different interventions. The main purpose of this study was to investigate the Clinical Frailty Scale (CFS) score as an independent predictor of short-, mid- and long-term mortality in critically ill older adults (aged ≥ 70) admitted to the emergency department (ED).

**Methods:**

This is a retrospective, single-center, observational study, involving critically ill older adults, recruited consecutively in an ED. All patients were followed for 6.5–7.5 years. The effect of CFS score on mortality was adjusted for the following confounders: age, sex, Charlson’s Comorbidity Index, individual comorbidities and vital parameters. All patients (*n* = 402) were included in the short- and mid-term analyses, while patients discharged alive (*n* = 302) were included in the long-term analysis. Short-term mortality was analysed with logistic regression, mid- and long-term mortality with log rank test and Cox proportional hazard models. The CFS was treated as a continuous variable in the primary analyses, and as a categorical variable in completing analyses.

**Results:**

There was a significant association between mortality at 30 days after ED admission and CFS score, adjusted OR (95% CI) 2.07 (1.64–2.62), *p* < 0.0001. There was a significant association between mortality at one year after ED admission and CFS score, adjusted HR (95% CI) 1.75 (1.53–2.01), *p* < 0.0001. There was a significant association between mortality 6.5–7.5 years after discharge and CFS score, adjusted HR (95% CI) 1.66 (1.46–1.89), *p* < 0.0001. Adjusted HRs are also reported for long-term mortality, when the CFS was treated as a categorical variable: CFS-score 5 versus 1–4: HR (95% CI) 1.98 (1.27–3.08); 6 versus 1–4: HR (95% CI) 3.60 (2.39–5.44); 7 versus 1–4: HR (95% CI) 3.95 (2.38–6.55); 8–9 versus 1–4: HR (95% CI) 20.08 (9.30–43.38). The completing analyses for short- and mid-term mortality indicated a similar risk-predictive value of the CFS.

**Conclusions:**

Clinical frailty scale score was independently associated with all-cause short-, mid- and long-term mortality. A nearly doubled risk of death was observed in frail patients. This information is clinically relevant, since individualised treatment and care planning for older adults should consider risk of death in different time perspectives.

**Supplementary Information:**

The online version contains supplementary material available at 10.1186/s12877-024-05463-7.

## Introduction

### Background

There is a large and growing population of older adults, including patients with complex needs [1]. This implies increasing healthcare needs, with an impact on healthcare [2]. A large increase in older adults in the emergency department (ED) and acute wards can be expected [3, 4]. As a group, older adults admitted to the ED and acute wards run a higher risk of death, ED revisits, hospitalizations, functional decline and loss of independence within a shorter period of time than younger patients [5, 6]. Older adults are characterized by the presence of age-related changes combined with acute and chronic diseases [4, 6, 7]. In many populations, frailty has been shown to be a strong risk factor for poor outcomes, especially short-term, such as increased dependence, hospitalization and death [8–10]. The frailty syndrome is associated with decreased physiological reserves and increased vulnerability [8, 9]. There are different frailty tools [11, 12]. In the Clinical Frailty Scale (CFS) an individual’s overall fitness or frailty is graded [13]. The scale score has a strong prognostic value regarding multiple outcomes and in different clinical contexts, particularly short- and midterm mortality [14–18].

The estimated prognosis influences the expected benefit/risk ratio of different interventions. The identification of risk predictors for mortality in older adults on admission to the ED and at subsequent discharge from hospital could provide clinically important information to support strategies to prevent future events and make individualized, informed treatment decisions [2, 4, 19]. Predictors of in-hospital and short-term mortality in older patients have been described in previous studies [2, 20–22]. In critically ill older adults in EDs and acute wards the evidence is scarce regarding the risk-predictive value of frailty, especially for long-term mortality [18].

### Aim

The aim of this study is to investigate the CFS score as a predictor of short-, mid- and long-term mortality in critically ill older adults (aged ≥ 70) admitted to the ED.

## Methods

### Study design and setting

This is a retrospective, observational, single-center cohort study. It includes critically ill older adults (≥ 70 years), recruited between February 2013 and February 2014 at the ED at the Northern Älvsborg-Uddevalla (NU) Hospital Group, a county hospital with an uptake population of 280 000 inhabitants, Region Västra Götaland, Sweden. The age cut off of 70 years was originally chosen as it is commonly used in similar studies. The collection of data was performed manually from paper-and virtual records by an experienced nurse and an experienced physician, both from the ED. Before any analyses were undertaken, the records of collected data were quality-checked and monitored by a senior statistician. A secondary data collection and analysis was performed regarding mortality until December 31, 2020. Some results of this analysis have been reported previously [22].

### Data collection and participants

A detailed description of the collection of primary data and mortality has been given previously [22]. All internal medicine ED patients were evaluated using the Rapid Emergency Treatment Triage System (RETTS) [23]. The RETTS relies on the following vital signs (VS): airway obstruction/stridor; oxygen saturation < 90%; respiratory rate < 8 or > 30 breaths per minute; regular heart rhythm > 130 or irregular heart rhythm > 150 beats per minute; systolic blood pressure < 90 mmHg; unconsciousness, defined as Reaction Level Scale (RLS) > 3 or Glasgow Coma Scale (GCS) < 8; ongoing seizure. Simultaneously the symptoms that caused the contact with health care is to be considered (the Emergency Signs and Symptoms code [ESS code]). The combination of VS and ESS gives the patient a colour of either red, orange, yellow, green or blue in order of severity of the condition and reflecting the time required to assessment by a physician. In this study we included patients given the colour red, reflecting urgent requirement of a physician assessment, i.e. critically ill patients.

Patients fulfilling the criteria of being critically ill were consecutively included. Patients were excluded if wrongly registered, if there was no written informed consent, or if there was cardiac arrest, need for acute percutaneous coronary intervention (PCI) or for the acute stroke fast track [24]. Retrospective collection of information was performed from the ambulance and hospital medical records. Mortality until December 31, 2020, was extracted from the State’s Personal Address Register (SPAR) at the Swedish Tax Agency [25]. Approximately 7% of all internal medicine patients ≥ 70 years of age admitted to the ED were considered critically ill.

Of 832 patients who were classified as critically ill, 610 gave written informed consent [24].

Of these, 402 patients were aged ≥ 70 years, and included in this current analysis. In August 2022, retrospective CFS rating of patients ≥ 70 years of age was performed by medical records review (EE supported by NE). The CFS assessment was based on the patient´s chronic health condition, approximately two weeks before the ED visit. There was a training on how to rate. The rating was done blinded to outcomes, but not to the hypotheses.

The Full Analysis Set (FAS) study population was defined as all included ED patients (aged ≥ 70) (*n* = 402). The Left Hospital Alive (LHA) study population was defined as all included patients discharged alive directly from the ED or from the index hospitalization (*n* = 302).

### Methods and measurements

The ambulance and medical records from the ED and the hospital medical wards were used for collection of patient characteristics and clinical variables. Age, sex, date and time of arrival at the ED, main symptoms and vital signs in the ambulance, working diagnosis in the ED and medical history including the Charlson Comorbidity Index (CCI) components were recorded. The type of department the patient was primarily hospitalized in, any care in the Intensive Care Unit (ICU) or cardiac Intensive Care Unit (cICU), total Length Of Stay (LOS) in hospital, diagnosis when leaving hospital and if the patient was dead or alive were recorded. Medical records were used for studying outcomes up to 12 months after discharge, including mortality, re-hospitalizations and total LOS. Another collection of mortality data was performed 6.5–7.5 years post-discharge, as described in the data collection section. The cases refer to unique individual patients, ie patients could only be included once.

For the follow-up of short- and mid-term mortality, all patients in the ED (Full Analysis Set (FAS), *n* = 402) were included. For the long-term follow-up 6.5–7.5 years, focus was set on patients who left hospital alive (Left Hospital Alive (LHA), *n* = 302). The long-term follow-up time was on December 31 2020, giving a range (one year) of follow-up time intervals depending on date of inclusion. All patients who left hospital alive were studied from discharge until death or until the end of the follow-up period.

#### The Clinical Frailty Scale (CFS-9)

The early identification of frail persons can initiate appropriate actions and support prognostication and risk stratification [11, 18, 19, 26–28]. Information on frailty status can promote the identification of persons in need of treatments across different health care sectors and with transdisciplinary acceptance [29–31].

The CFS combines the assessment of disability, comorbidity and cognitive status. In a recent study regarding the Swedish CFS version, the inter-rater reliability was excellent [29]. Several studies have indicated that retrospective scoring of the CFS, based on patient charts, is associated with a risk-predictive value comparable with that derived from bedside assessment [32–35].

#### The Charlson Comorbidity Index (CCI)

Each person’s total burden of morbidity was assessed by the CCI [36, 37]. The CCI consists of 17 components of comorbidity and it predicts mortality for a patient. Each component is assigned a score of 1, 2, 3, or 6, depending on the risk of death.

### Outcomes

All-cause short-term mortality within 30 days after admission to the ED (FAS population).

All-cause mid-term mortality within one year after admission to the ED (FAS population).

All-cause post-discharge long-term mortality until December 31, 2020 (6.5–7.5 years) (LHA population).

### Statistical analysis

Continuous variables are reported as mean (SD)/median (Q1;Q3), and categorical variables as numbers and percentages.The purpose of the main analyses is to study the effect of CFS on mortality adjusted for relevant confounders in the baseline characteristics. Short time mortality (30 days) of the FAS population is analysed as dichotomous outcome with logistic regression. Results are reported as Odds Ratio (OR) (univariable) and adjusted OR (aOR) (multivariable) with 95% confidence interval (CI) and Area Under the Receiver Operating Characteristic (ROC) curve (Area Under Curve; AUC). Logistic regression analysis is performed for each independent variable to predict the outcome. Area Under ROC-curve (AUC-statistics) was calculated for description of goodness of predictors. Mid-term mortality (one year) of the FAS population is analysed as time to death with log-rank test for univariable *p*-values and Cox proportional Hazard model to estimate Hazard Ratio (HR) with 95% CI, both unadjusted and adjusted. Results are reported as HR and adjusted HR (aHR) with 95% CI and *p*-values and the Uno’s Concordance Index (Uno C-index). The Uno C-index is interpreted in the same way as AUC. Within each subgroup mid-mortality is reported as event rates per 10 observation years. Long-term mortality (6.5–7.5 years) of the LHA population is analysed as time to death with log-rank test for univariable *p*-values and Cox proportional Hazard model to estimate HRs with 95% CI both unadjusted and adjusted. Results are reported as HRs and adjusted HRs (aHRs) with 95% CI, *p*-values and the Uno C-index. Within each subgroup mid-mortality is reported as event rates per 10 observation years. The Kaplan–Meier (KM) method is used to calculate cumulative mortality curves, using 100—KM survival estimate. In the primary analyses, the CFS is treated as a continuous (ordered categorical) variable, in order not to underestimate the risk-predictive value of the higher CFS-levels.

The proportional hazards assumption was tested using martingale residuals and was met for the long-term analyses both when CFS was used as a linear variable and as a categorical variable. For the mid-term analyses the proportional hazards assumption was not met when CFS was used as a linear variable. When CFS was used as a categorical variable, the proportional hazards assumption was met except for CFS 5 vs CFS 1–4. In these cases, the Hazard ratio (HR) is still interpretable as a population-average hazard ratio over the follow-up period. Analyses for the three time-perspectives (short-, mid- and long-term), using CFS as a categorical variable have been added. Due to low numbers with CFS 1–3 and CFS 9, we have categorized patients into CFS 1–4,5,6,7 and 8–9. In the analyses CFS 1–4 was used as reference and compared with 5,6,7 and 8–9 respectively.

The CCI was analysed as a continuous variable. All tests are two-tailed and conducted at 0.05 significance level. All analyses are performed using SAS® v9.3 (Cary, NC). None of the statistical methods used assume normality. There were no extreme values. Age was the only continuous variable.

## Results

**Fig. 1:**
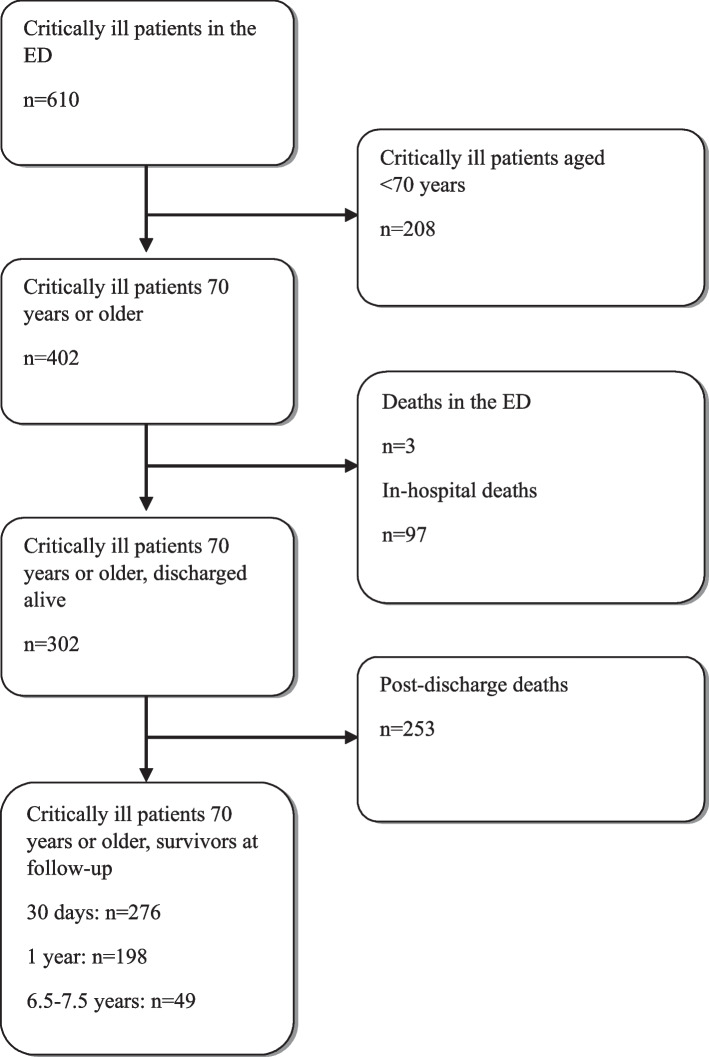
Flow chart

Of 610 critically ill patients included in the ED, 402 were aged ≥ 70 years. Three of these patients (0.7%) died in the ED and six (1.5%) returned home directly from the ED. There were 97 (24.1%) in-hospital deaths. Of the 302 patients discharged alive, directly from the ED or from a hospital ward, 253 (83.8%) died before the end of final follow-up (on December 31, 2020), see flow chart, Fig. 1**.**

### Baseline characteristics

The baseline characteristics of all patients aged ≥ 70 years are shown in Table 1. The mean age of the FAS population was 82.6 years (SD 6.3) and 220 (54.7%) were male. The patients had a large comorbidity burden. The most commonly reported main symptoms on admission were dyspnoea, an episode of unconsciousness, chest pain, seizure and vomiting. In the FAS-population, 100 patients (24,9%) were classified as CFS-levels 1–4 (robust or very mildly frail), 177 patients (44,0%) as CFS-levels 5–6 (mildly or moderately frail), and 125 patients (31.1%) as CFS-levels 7–9 (severely frail or terminally ill). In the LHA population, 91 patients (30.1%) were classified as CFS 1–4, 142 (47.0%) patients as CFS 5–6, and 69 (22.8%) patients as CFS 7–9.

**Table 1:** Baseline characteristics of critically ill patients aged ≥70 years admitted to the ED

**Variable, *n (%)***	**All patients (*n=*402)**	**Discharged alive (***n=***302)**
**Demografic characteristics **		
Age	82.6 (6.3)	82.3 (6.2)
Female	182 (45.3%)	138 (45.7%)
CFS-score		
1	1 (0.25%)	1 (0.3%)
2	1 (0.25%)	1 (0.3%)
3	16 (4.0%)	14 (4.6%)
4	82 (20.4%)	75 (24.8%)
5	71 (17.7%)	55 (18.2%)
6	106 (26.4%)	87 (28.8%)
7	79 (19.7%)	53 (17.6%)
8	31 (7.7%)	11 (3.6%)
9	15 (3.7%)	5 (1.7%)
**CCI-variables**		
CCI-score		
0	68 (16.9%)	52 (17.2%)
1-2	208 (51.7%)	156 (51.7%)
3-4	86 (21.4%)	65 (21.5%)
>4	40 (10.0%)	29 (9.6%)
Previous MI	81 (20.2%)	58 (19.3%)
CHF	92 (22.9%)	70 (23.2%)
PAD	28 (7.0%)	19 (6.3%)
CVD	91 (22.6%)	70 (23.2%)
Dementia	70 (17.4%)	50 (16.6%)
COPD	88 (21.9%)	72 (23.8%)
Diabetes without complications	73 (18.2%)	55 (18.2%)
Diabetes with complications	19 (4.7%)	16 (5.3%)
Moderate to severe CKD	36 (9.0%)	29 (9.6%)
Tumor without metastases	26 (6.5%)	18 (6.0%)
Tumor with metastases	11 (2.7%)	7 (2.3%)
Lymphoma	6 (1.5%)	5 (1.7%)
Leukemia	1 (0.2%)	0 (0.0%)
**Main symptom on admission**		
Dyspnea	200 (49.8%)	148 (49.0%)
Unconsciousness	59 (14.7%)	33 (10.9%)
Chest pain	46 (11.4%)	39 (12.9%)
Seizure attack	16 (4.0%)	15 (5.0%)
Vomiting	22 (5.5%)	19 (6.3%)
**Vital parameters on admission**		
Obstructive airway	26 (6.5%)	17 (5.6%)
Hypoxia^a^	226 (56.9%)	153 (51.5%)
Hypotension^b^	51 (12.8%)	39 (13.0%)
Respiration rate (breaths/min) *≤* 8 or ≥ 30	185 (51.5%)	129 (48.3%)
Heart rate (bpm), ≥130 OR ≥ 150^c^	66 (16.5%)	57 (18.9%)
RLS>3	65 (16.2%)	38 (12.6%)
Ongoing seizures	15 (3.7%)	13 (4.3%)
Signs of infection	141 (35.1%)	111 (36.8%)
**Discharge diagnosis**		
Pneumonia	85 (21.1%)	63 (20.9%)
Heart Failure	33 (8.2%)	26 (8.6%)
Atrial fibrillation	21 (5.2%)	20 (6.6%)
COPD	27 (6.7%)	19 (6.3%)
Urosepsis	29 (7.2%)	23 (7.6%)
**Hospital care level**		
Intensive care unit or cardiac intensive care unit	76 (18.9%)	59 (19.5%)
Medical emergency ward	130 (32.3%)	101 (33.4%)
Other wards	187 (46.5%)	136 (45.0%)
Not Hospitalized	9 (2.2%)	6 (2.0%)

For categorical variables n (%) is presented

For continuous variables Mean (SD) / Median (Min; Max) is presented

*CFS* Clinical frailty scale, *CCI* Charlson Comorbidity Index, *MI* Myocardial Infarction, *CHF* Congestive Heart Failure, *PAD* Peripheral Arterial Disease, *CVD* Cerebrovascular disease, *COPD* Chronic Obstructive Pulmonary Disease, *CKD* Chronic Kidney Disease, *BPM* Beats per minute; RLS, Reaction Level Scale

Three patients died in the ED, and six patients could return home directly from the ED

^a^Oxygen saturation <90%

^b^Oxygen saturation <90%

^c^Regular/irregular

### Short-term mortality (*n* = 402)

#### Unadjusted analyses

The association between baseline characteristics and all-cause mortality until 30 days after admission to the ED is presented as unadjusted ORs in Additional File 1. There were 126 deaths. The CFS was significantly associated with mortality: CFS 1–4, 10 deaths (10%); CFS 5–6, 41 deaths (23.2%); CFS 7–9, 75 deaths (60.0%), OR (95% CI) 2.10 (1.74–2.53), *p* < 0.0001, AUC 0.76 (0.70–0.81). The following variables were also significantly associated with 30-day mortality: age, dementia, Chronic Obstructive Pulmonary Disease (COPD), hypoxia on admission, unconsciousness on admission and tachycardia (all *p* < 0.05).

#### Adjusted analyses

In multivariable analyses, when treated as a continuous variable, the CFS was significantly associated with mortality, adjusted OR (95% CI) 2.07 (1.64–2.62), *p* < 0.0001. Table 2. Adjusted ORs are also reported for analyses when the CFS was treated as a categorical variable: CFS-score 5 versus 1–4: OR (95% CI) 3.34 (1.27–8.77); 6 versus 1–4: OR (95% CI) 2.33 (0.95–5.73); 7 versus 1–4: OR (95% CI): 6.47 (2.42–17.31); 8–9 versus 1–4: OR (95% CI) 51.03 (14.70–177.19).

**Table 2:** Adjusted analyses regarding all-cause short-, mid- and long-term mortality

All-cause mortality within 30 days after ED admission (*n*=402)			
	Odds ratio	95% CI	*P*-value
CFS-score (continuous)	2.07	1.64-2.62	<.0001
CFS-score (categorical)			
5 versus 1-4	3.34	1.27-8.77	0.014
6 versus 1-4	2.33	0.95-5.73	0.065
7 versus 1-4	6.47	2.42-17.31	0.0002
8-9 versus 1-4	51.03	14.70-177.19	<.0001
All-cause mortality until one year after ED admission (n=402)			
	Hazard ratio	95% CI	*P*-value
CFS-score (continuous)	1.75	1.53-2.01	<.0001
CFS-score (categorical)			
5 versus 1-4	2.76	1.47-5.20	0.0016
6 versus 1-4	3.25	1.84-5.76	1.84-5.76
7 versus 1-4	5.75	3.09-10.71	<.0001
8-9 versus 1-4	14.66	7.63-28.18	<.0001
All-cause mortality 6.5-7.5 years after discharge (n=302)			
	Hazard ratio	95% CI	*P*-value
CFS-score (continuous)	1.66	1.46-1.89	<.0001
CFS-score (categorical)			
5 versus 1-4	1.98	1.27-3.08	0.0025
6 versus 1-4	3.60	2.39-5.44	<.0001
7 versus 1-4	3.95	2.38-6.55	<.0001
8-9 versus 1-4	20.08	9.30-43.38	<.0001

In the reported primary analyses, the CFS was treated as a continuous variable

In sensitivity analyses, the CFS was treated as a categorical variable with CFS-score 1-4 as reference

Short time mortality (30 days) is analysed with logistic regression

Mid-term mortality (one year) is analysed with a Cox proportional hazard model

Long-term mortality (6.5-7.5 years) is analysed with a Cox proportional Hazard model

Confounders in the analyses: age, sex, CCI-score, previous MI, CHF, PAD, CVD, dementia, COPD, diabetes, moderate to severe CKD, tumour, lymphoma, obstructive airway, hypoxia, hypotension, heart rate (bpm) ≥130 OR ≥ 150, RLS > 3, ongoing seizures, signs of infection

Hypoxia=Oxygen saturation <90%

Hypotension=Systolic blood pressure <90 mmHg

Heart rate: regular ≥130; irregular ≥ 150

*CFS* Clinical frailty scale, *CCI* Charlson Comorbidity Index, *MI* Myocardial Infarction, *CHF* Congestive Heart Failure, *PAD* Peripheral Arterial Disease, *CVD* Cerebrovascular disease, *COPD* Chronic Obstructive Pulmonary Disease, *CKD* Chronic Kidney Disease, *BPM* beats per minute, *RLS* Reaction Level Scale, *OR* odds ratiodney Disease, *BPM* beats per minute, *RLS* Reaction Level Scale, *OR* odds ratio

### Mid-term mortality (*n* = 402)

#### Unadjusted analyses

The association between baseline characteristics and all-cause mortality until one year after admission to the ED is presented as unadjusted HRs in Additional File 2. There were 204 deaths. The CFS was significantly associated with mortality: CFS 1–4, 17 deaths (17.0%); CFS 5–6, 86 deaths (48.6%); CFS 7–9, 101 deaths (80.8%), HR (95% CI) 1.74 (1.57- 1.93), *p* < 0.0001, Uno-C index 0.71. The following variables were also significantly associated with one-year-mortality: age, CCI, dementia, metastatic tumour, unconsciousness on admission and hypoxia on admission (all *p* < 0.05). Kaplan–Meier (KM) survival curves (one year) versus CFS-levels are presented in Additional File 3.

#### Adjusted analyses

In adjusted analyses, the CFS was significantly associated with mortality, adjusted HR (95% CI) 1.75 (1.53–2.01), *p* < 0.0001. Table 2. Adjusted HRs are also reported for analyses when the CFS was treated as a categorical variable: CFS-score 5 versus 1–4: HR (95% CI) 2.76 (1.47–5.20); 6 versus 1–4: HR (95% CI) 3.25 (1.84–5.76); 7 versus 1–4: HR (95% CI) 5.75 (3.09–10.71); 8–9 versus 1–4: HR (95% CI) 14.66 (7.63–28.18).

### Long-term mortality (*n* = 302)

#### Unadjusted analyses

**Fig. 2:**
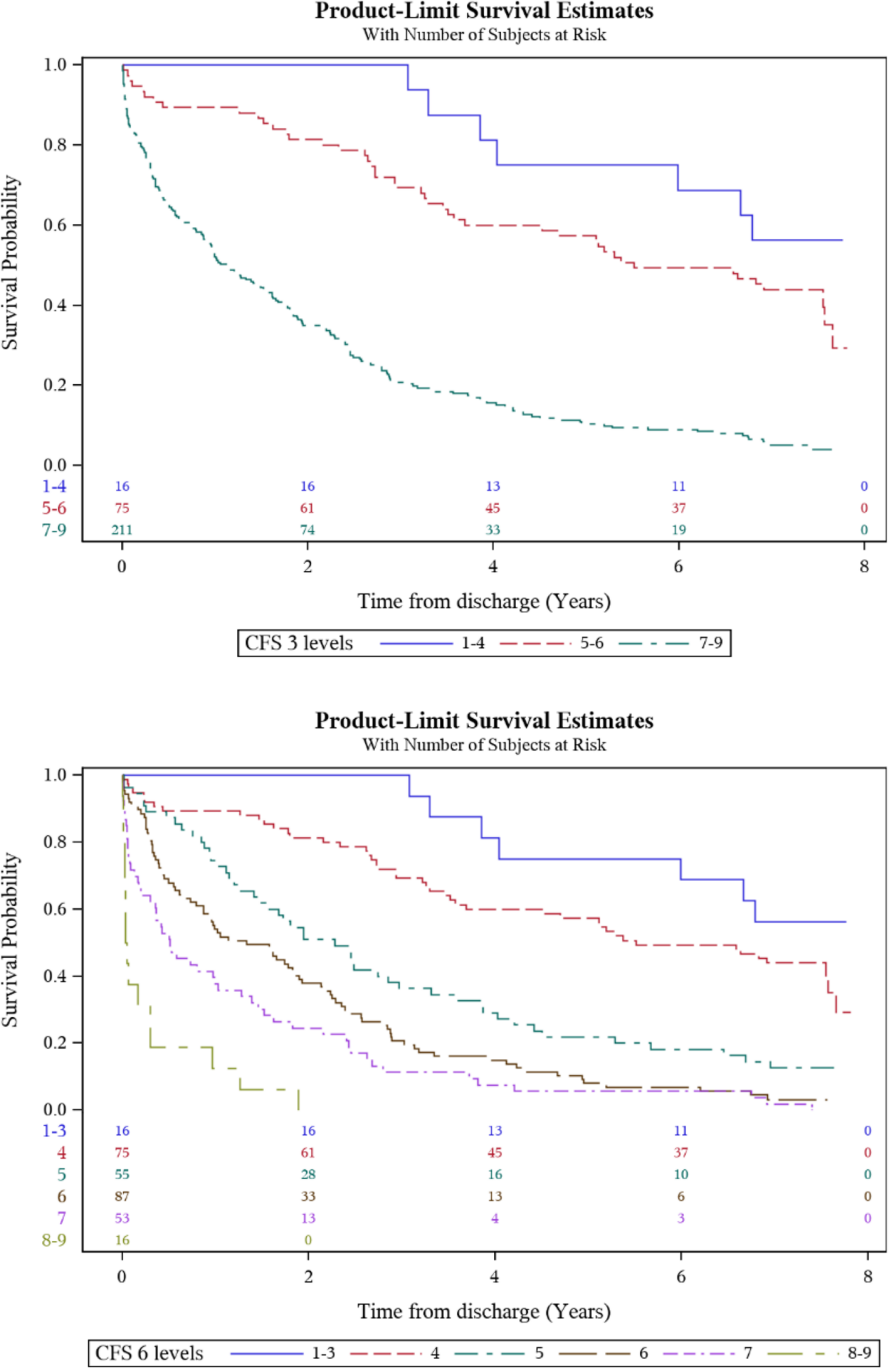
Kaplan–Meier (KM) survival curves (long-term) versus CFS-levels

The association between baseline characteristics and all-cause mortality 6.5–7.5 years after discharge (until December 31, 2020) is presented as unadjusted HRs in **Additional file 4**. There were 253 post-discharge deaths. The CFS was significantly associated with mortality: CFS 1–4, 52 deaths (57.1%); CFS 5–6, 132 deaths (93.0%); CFS 7–9, 69 deaths (100.0%), HR (95% CI) 1.80 (1.62–1.99), *p* < 0.0001, Uno-C index 0.71. The following variables were also significantly associated with long-term-mortality: age, CCI, Chronic Heart Failure (CHF), CardioVascular Disease (CVD), dementia, Chronic Kidney Disease (CKD), metastatic tumour and hypoxia on admission (all *p* < 0.05). Kaplan–Meier (KM) survival curves (long-term) versus CFS-levels are presented in Fig. 2**.**

#### Adjusted analyses

In adjusted analyses, the CFS was significantly associated with mortality, adjusted HR (95% CI) 1.66 (1.46–1.89), *p* < 0.0001.Table 2. Adjusted HRs are also reported for analyses when the CFS was treated as a categorical variable: CFS-score 5 versus 1–4: HR (95% CI) 1.98 (1.27–3.08); 6 versus 1–4: HR (95% CI) 3.60 (2.39–5.44); 7 versus 1–4: HR (95% CI) 3.95 (2.38–6.55); 8–9 versus 1–4: HR (95% CI) 20.08 (9.30–43.38).

## Discussion

This study demonstrates that frailty, assessed by CFS scores, adds important short-, mid- and long-term prognostic information for critically ill older adults admitted to the emergency department. Frailty, assessed with the CFS, was independently associated with all-cause mortality, with curves diverging early and remaining separated over 6.5–7.5 years follow-up. The CFS scores association with risk of death remained significant after extensive adjustment for other prognostic factors, such as age, sex, comorbidity burden captured by both CCI and individual diagnoses, and vital signs on admission. A nearly doubled risk of death was observed in frail patients.

The analysis of long-term mortality was based on a follow-up of 6.5–7.5 years post-discharge via a state agency register. Different time perspectives were applied in a clinically relevant way for both short-, mid- and long-term mortality. Individualized care planning should take account of risk prediction in different time perspectives. Whereas the benefits of preventive interventions accumulate over time, the potential adverse effects of many interventions are immediate. Therefore, it is reasonable that clinical decision-making and priorities to some extent vary with life expectancy. In the primary analyses we treated the CFS as a continuous (ordered categorical) variable with all nine levels intact in order not to underestimate the risk-predictive value of the higher CFS levels. The use of the scale in such a granular way seems to be in line with previous recommendations [38]. In completing analyses the CFS was treated as a categorical variable with similar results.

We chose logistic regression for the short-term follow-up (30 days) to make comparisons with previous reports easier as most 30 days studies have reported logistic regression. This was appropriate as we had complete follow-up over 30 days, without any censored cases. For longer term follow-up Cox regression, with a time to event analysis, was the most reasonable choice as we had censored cases. It is important to include all patients in the short-term follow-up because a significant proportion of the events occurred early. Nevertheless, long-term outcome in patients surviving the acute phase is important for long-term treatment and planning.

The prognostic value of the CFS scores harmonizes with the results of previous studies [10, 18, 27, 38]. Furthermore, our results indicate that the prognostic impact of the CFS scores remains for critically ill older adults over time, also regarding long-term mortality. Previous studies have established hypoxia as a strong risk factor in the emergency care context. Even a moderate decrease in oxygen levels in the emergency care has been reported to have an impact on the prognosis [39]. However, it can be discussed whether the possible correlation in our analyses between hypoxia and other included variables, such as COPD and CHF, might result in multicollinearity in the regression analyses. The inclusion of additional relevant factors in the analyses was considered e.g. Glasgow Coma Scale (GCS) score and history of fall. However, we did include many covariates, the number approaching what could be considered as an upper limit from the statistical point of view considering the number of events.

The CFS has good sensitivity, specificity and predictive validity [9], and there is evidence of the ability of the scale to predict different adverse health outcomes, especially mortality [14–18]. In different guidelines, both geriatric and non-geriatric, frailty assessment is recommended, e.g. regarding cardiovascular care [12, 40]. A valid, reliable and easily applied measurement of frailty might have the potential to facilitate communication and collaboration in the diagnostics, treatment and rehabilitation of frail patients within as well as across health-care sectors [41]. This includes the use of the CFS for critically ill older adults admitted to the emergency ward. However, it should be emphasized, that there is a need for prospective interventional studies in different clinical contexts, where the potential of the CFS scores to guide specific interventions is explored. Studies evaluating models for risk prediction where the CFS is combined with other factors to create an even more effective tool for risk prediction could also be considered.

### Strengths and limitations

Our study has several strengths. Critically ill elderly patients in a common ED population were recruited consecutively. Survival information at follow-up was complete for all but one patient, based on a reliable source of mortality data. It is a strength that three different and clinically relevant time perspectives were applied. The analyses included a very long follow-up of mortality data up to 7.5 years post-discharge, which to our knowledge is very rare regarding this patient population. In order to assess frailty, we used the CFS, a reliable frailty instrument, which can be easily applied in clinical practice.

There are some limitations and points to discuss. The patient recruitment of the study was performed in 2013–2014, which might look as a long time ago given the rapid advancements in medical practice, but this time is necessary to allow a long-term follow-up. Patients included in the stroke fast track and the PCI pathway were not included in this study, as they have separate pathways into the hospital thus bypassing the ED. However, the majority of all patients with acute cerebrovascular disease or acute coronary syndrome came via the ED, and were thus possible to include. The size of the study population was moderate, but when it comes to critically ill older adults, 402 consecutively recruited patients could be regarded as a study sample of reasonable size. The scoring of the CFS was done retrospectively, which might be considered as a source of loss of precision and quality of the scoring. However, several studies have indicated that retrospective scoring of the CFS is associated with a risk-predictive value comparable with that derived from bedside scoring [32–35]. The CFS rater was blinded regarding the outcomes for the patients. For the mid-term analyses the proportional hazards assumption was not met when CFS was used as a linear variable. When CFS was used as a categorical variable, the proportional hazards assumption was met except for CFS 5 vs CFS 1–4. The result from the mid-term analysis has to be interpreted with caution. However, in these cases, the HR is still interpretable as a population-average HR over the follow-up period. We have data from admission to 30 days follow-up and from discharge to long-term follow-up, with similar prognostic value of CFS, overlapping the results from the mid-term follow-up. Therefore, we believe it is reasonable to report all three models.

## Conclusion

Clinical frailty scale score was independently associated with all-cause short-, mid- and long-term mortality for critically ill older adults admitted to the emergency department. The impact of CFS scores on risk remained significant after extensive adjustment for other prognostic factors. A nearly doubled risk of death was observed in frail patients. This information is clinically relevant, since individualized care planning for older adults should take account of risk prediction in different time perspectives.

## Supplementary Information

Additional file 1 Unadjusted analysis regarding all-cause mortality until 30 days after admission to the ED

Additional file 2 Unadjusted analysis regarding all-cause mortality until one year after admission to the ED

Additional file 3 Kaplan-Meier (KM) survival curves (one year) versus CFS-levels

Additional file 4 Unadjusted analysis regarding all-cause mortality 6.5-7.5 years after discharge

## Data Availability

The datasets used and/or analysed during the current study are available from the corresponding author on reasonable request.

## Abbreviations

AUC: Area under curve
CFS: Clinical Frailty Scale
CCI: Charlson Comorbidity Index
CGA: Comprehensive geriatric assessment
COPD: Chronic obstructive pulmonary disease
ED: Emergency department
FI: Frailty index
HR: Hazard ratio
ICU: Intensive care unit
KM: Kaplan–Meier
LOS: Length of stay
MEC: Medical Emergency Care
OR: Odds ratio
PCI: Percutaneous coronary intervention
RETTS: Rapid Emergency Treatment Triage System
RLS: Reaction Level Scale
ROC: Receiver Operating Characteristic
SPAR: State’s Personal Address Register
WHO: World Health Organization
