# Supplementary material for: Clinical Frailty Scale score is a predictor of short-, mid- and long-term mortality in critically ill older adults (≥ 70 years) admitted to the emergency department: an observational study
Source: BMC Geriatr. 2024 Oct 21;24:852. doi: 10.1186/s12877-024-05463-7 (PMC11492669; doi:10.1186/s12877-024-05463-7)
Supplement: Supplementary file 1 — Additional file 1 Unadjusted analysis regarding all-cause mortality until 30 days after admission to the ED [file 12877_2024_5463_MOESM1_ESM.docx]

| **Additional File 1** Unadjusted analysis regarding all-cause mortality until 30 days after admission to the ED | | | | |
| --- | --- | --- | --- | --- |
| **Variable** | **Value** | **n (%) of event** | **OR (95%CI) Deaths within 30 days** | **P-value** |
| CFS-score (continuous) | 1-4 | 10 (10.0) |  |  |
|  | 5-6 | 41 (23.2) |  |  |
|  | 7-9 | 75 (60.0) | 2.10 (1.74-2.53) | <0.0001 |
| CFS-score (categorical)  5 versus 1-4  6 versus 1-4  7 versus 1-4  8 versus 1-4 |  |  | 3.06 (1.31-7.11)  2.49 (1.12-5.55)  7.54 (3.42-16.59)  50.14 (17.79-141.35) | 0.0095  0.025  <.0001  <.0001 |
| Age (OR per 5 units) | 69.7-79.5 | 31 (23.1) |  |  |
|  | 79.5-85.7 | 46 (34.3) |  |  |
|  | 85.7-99.6 | 49 (36.6) | 1.34 (1.13-1.59) | 0.0010 |
| Sex | Female | 55 (30.2) |  |  |
|  | Male | 71 (32.3) | 1.10 (0.72-1.68) | 0.66 |
| **CCI-variables** | | | | |
| CCI-score | 0-<2 | 52 (28.7) |  |  |
|  | 2-<3 | 32 (33.7) |  |  |
|  | 3-12 | 42 (33.3) | 1.09 (0.97-1.23) | 0.13 |
| Previous MI | No | 98 (30.6) |  |  |
|  | Yes | 28 (34.6) | 1.20 (0.71-2.00) | 0.49 |
| CHF | No | 100 (32.3) |  |  |
|  | Yes | 26 (28.3) | 0.83 (0.50-1.38) | 0.47 |
| PAD | No | 116 (31.0) |  |  |
|  | Yes | 10 (35.7) | 1.24 (0.55-2.76) | 0.61 |
| CVD | No | 96 (30.9) |  |  |
|  | Yes | 30 (33.0) | 1.10 (0.67-1.81) | 0.70 |
| Dementia | No | 92 (27.7) |  |  |
|  | Yes | 34 (48.6) | 2.46 (1.45-4.17) | 0.0008 |
| COPD | No | 108 (34.4) |  |  |
|  | Yes | 18 (20.5) | 0.49 (0.28-0.87) | 0.014 |
| Diabetes Mellitus 0/1/2 | No | 102 (32.9) |  |  |
|  | Without Complications | 20 (27.4) |  |  |
|  | With Complications | 4 (21.1) | 0.75 (0.50-1.14) | 0.18 |
| Moderate to severe CKD | No | 115 (31.4) |  |  |
|  | Yes | 11 (30.6) | 0.98 (0.68-1.42) | 0.92 |
| Tumor 0/1/2 | No | 110 (30.1) |  |  |
|  | Without Metastases | 10 (38.5) |  |  |
|  | Metastatic | 6 (54.5) | 1.19 (0.99-1.43) | 0.066 |
| Lymphoma | No | 125 (31.6) |  |  |
|  | Yes | 1 (16.7) | 0.66 (0.22-1.94) | 0.45 |
| Leukemia* | No | 125 (31.2) |  |  |
|  | Yes | 1 (100.0) | + infinity | 0.63 |
| **Vital signs on admission** | | | | |
| Obstructive airway | No | 115 (30.6) |  |  |
|  | Yes | 11 (42.3) | 1.66 (0.74-3.74) | 0.22 |
| Hypoxia | No | 34 (19.9) |  |  |
|  | Yes | 92 (40.7) | 2.77 (1.75-4.38) | <0.0001 |
| Hypotension 2 | No | 111 (31.9) |  |  |
|  | Yes | 14 (27.5) | 0.81 (0.42-1.56) | 0.52 |
| Respiratory rate (breaths/min) ≤ 8 or ≥ 30 | No | 47 (27.0) |  |  |
|  | Yes | 70 (37.8) | 1.64 (1.05-2.57) | 0.029 |
| Heart rate (bpm), ≥130 OR ≥ 1503 | No | 115 (34.3) |  |  |
|  | Yes | 11 (16.7) | 0.38 (0.19-0.76) | 0.0060 |
| RLS > 3 | No | 92 (27.3) |  |  |
|  | Yes | 34 (52.3) | 2.92 (1.70-5.02) | 0.0001 |
| Ongoing seizures | No | 124 (32.0) |  |  |
|  | Yes | 2 (13.3) | 0.33 (0.07-1.47) | 0.14 |
| All tests are performed with univariable logistic regression.  P-values, OR and Area under ROC-curve are based on original values and not on stratified groups.  OR is the ratio for the odds for an increase of the predictor of one unit.  Oxygen saturation <90%  2 Systolic blood pressure <90 mmHg  3 Regular ≥130 or irregular ≥ 150  Analyses reported both with CFS treated as a continuous and as a categorical variable, respectively.  *Only one patient had leukemia  Missing: Previous MI n=1, Hypoxia n=5, Hypotension n=3, Respiration rate n=43, Heart rate n=1  CFS, Clinical frailty scale; CCI, Charlson Comorbidity Index; MI, Myocardial Infarction; CHF, Congestive Heart Failure; PAD, Peripheral Arterial Disease; CVD, Cerebrovascular disease; COPD, Chronic Obstructive Pulmonary Disease; CKD, Chronic Kidney Disease; BPM, beats per minute; RLS, Reaction Level Scale; BPM, beats per minute | | | | |
