# Supplementary material for: Clinical Frailty Scale score is a predictor of short-, mid- and long-term mortality in critically ill older adults (≥ 70 years) admitted to the emergency department: an observational study
Source: BMC Geriatr. 2024 Oct 21;24:852. doi: 10.1186/s12877-024-05463-7 (PMC11492669; doi:10.1186/s12877-024-05463-7)
Supplement: Supplementary file 2 — Additional file 2 Unadjusted analysis regarding all-cause mortality until one year after admission to the ED [file 12877_2024_5463_MOESM2_ESM.docx]

| **Additional file 2.** Unadjusted analysis regarding all-cause mortality until one year after admission to the ED | | | | | | | |
| --- | --- | --- | --- | --- | --- | --- | --- |
| **Variable** | **Value** | **n (%) of event** | **Total time (Years)** | **Events per 10 Years (95% CI)** | **Log-Rank p-value** | **HR (95% CI)** | **Cox p-value** |
| Age | 69.7-79.5 | 52 (38.8) | 93.5 | 5.35 (4.04-7.07) |  |  |  |
|  | 79.5-85.7 | 68 (50.7) | 75.9 | 8.82 (6.93-11.24) |  |  |  |
|  | 85.8-99.6 | 84 (62.7) | 64.5 | 12.72 (10.22-15.82) | <.0001 | 1.06 (1.03-1.08) | <.0001 |
| Sex | Female (ref.) | 91 (50.0) | 108.3 | 8.03 (6.50-9.92) |  |  |  |
|  | Male | 113 (51.4) | 125.6 | 8.92 (7.40-10.74) | 0.79 | 1.04 (0.79-1.37) | 0.79 |
| CFS-score (continuous) | 1-4 | 17 (17.0) | 85.0 | 2.00 (1.24-3.24) |  |  |  |
|  | 5-6 | 86 (48.6) | 113.6 | 7.22 (5.80-8.98) |  |  |  |
|  | 7-9 | 101 (80.8) | 35.4 | 28.28 (23.20-34.47) | <.0001 | 1.74 (1.57-1.93) | <.0001 |
| CFS-score (categorical)  5 versus 1-4 2.92 (1.61-5.30) 0.0004  6 versus 1-4 3.75 (2.18-6.45) <.0001  7 versus 1-4 6.83 (3.97-11.75) <.0001  8 versus 1-4 16.62 (9.35-29.54) <.0001  **CCI-variables** | | | | | | | |
| CCI-score | 0-1 | 78 (43.1) | 113.3 | 6.53 (5.19-8.21) |  |  |  |
|  | 2-2 | 56 (58.9) | 53.8 | 10.41 (7.98-13.57) |  |  |  |
|  | 3-12 | 70 (55.6) | 66.8 | 10.33 (8.14-13.11) | 0.045 | 1.11 (1.03-1.19) | 0.005 |
| Previous MI | No (ref.) | 160 (50.0) | 188.3 | 8.34 (7.13-9.76) |  |  |  |
|  | Yes | 44 (54.3) | 44.7 | 9.41 (6.92-12.79) | 0.44 | 1.14 (0.82-1.59) | 0.45 |
| CHF | No (ref.) | 151 (48.7) | 181.8 | 8.03 (6.82-9.45) |  |  |  |
|  | Yes | 53 (57.6) | 52.1 | 10.17 (7.74-13.36) | 0.28 | 1.19 (0.87-1.62) | 0.29 |
| PAD | No (ref.) | 190 (50.8) | 218.0 | 8.49 (7.34-9.81) |  |  |  |
|  | Yes | 14 (50.0) | 15.9 | 8.79 (5.08-15.21) | 0.90 | 1.04 (0.60-1.78) | 0.90 |
| CVD | No (ref.) | 153 (49.2) | 184.7 | 8.01 (6.82-9.42) |  |  |  |
|  | Yes | 51 (56.0) | 49.3 | 10.35 (7.84-13.67) | 0.31 | 1.18 (0.86-1.62) | 0.31 |
| Dementia | No (ref.) | 155 (46.7) | 204.9 | 7.37 (6.28-8.65) |  |  |  |
|  | Yes | 49 (70.0) | 29.0 | 16.55 (12.41-22.08) | 0.0003 | 1.78 (1.29-2.46) | 0.0005 |
| COPD | No (ref.) | 165 (52.5) | 173.6 | 9.21 (7.89-10.77) |  |  |  |
|  | Yes | 39 (44.3) | 60.3 | 6.47 (4.70-8.89) | 0.087 | 0.74 (0.52-1.05) | 0.091 |
| Diabetes Mellitus 0/1/2 | No (ref.) | 160 (51.6) | 178.1 | 8.76 (7.48-10.26) |  |  |  |
|  | Without Complications | 36 (49.3) | 43.2 | 8.11 (5.79-11.35) |  | 0.92 (0.64-1.32) | 0.65 |
|  | With Complications | 8 (42.1) | 12.7 | 6.30 (3.00-13.23) | 0.56 | 0.74 (0.36-1.50) | 0.40 |
| Moderate to severe CKD | No (ref.) | 183 (50.0) | 214.9 | 8.28 (7.15-9.60) |  |  |  |
|  | Yes | 21 (58.3) | 19.1 | 11.01 (7.07-17.14) | 0.47 | 1.18 (0.75-1.85) | 0.47 |
| Tumor 0/1/2 | No (ref.) | 180 (49.3) | 217.5 | 8.04 (6.93-9.33) |  |  |  |
|  | Without Metastases | 15 (57.7) | 13.3 | 11.29 (6.63-19.21) |  | 1.22 (0.72-2.07) | 0.45 |
|  | Metastatic | 9 (81.8) | 3.1 | 28.92 (13.76-60.79) | 0.49 | 2.34 (1.20-4.57) | 0.013 |
| Lymphoma | No (ref.) | 201 (50.8) | 230.6 | 8.50 (7.39-9.78) |  |  |  |
|  | Yes | 3 (50.0) | 3.4 | 8.92 (2.02-39.34) | 0.89 | 0.93 (0.30-2.89) | 0.89 |
| Leukemia* | No (ref.) | 203 (50.6) | 233.9 | 8.46 (7.36-9.73) |  |  |  |
|  | Yes | 1 (100.0) | 0.0 | (-) | 0.027 | 6.64 (0.92-47.92) | 0.061 |
| **Vital signs on admission** | | | | | | | |
| Obstructive airway | No (ref.) | 191 (50.8) | 219.9 | 8.51 (7.37-9.82) |  |  |  |
|  | Yes | 13 (50.0) | 14.1 | 8.52 (4.69-15.45) | 0.67 | 1.13 (0.64-1.98) | 0.67 |
| Hypoxia | No (ref.) | 58 (33.9) | 125.7 | 4.45 (3.42-5.80) |  |  |  |
|  | Yes | 144 (63.7) | 104.4 | 13.50 (11.44-15.94) | <.0001 | 2.44 (1.79-3.31) | <.0001 |
| Hypotension2 | No (ref.) | 177 (50.9) | 201.5 | 8.54 (7.35-9.92) |  |  |  |
|  | Yes | 25 (49.0) | 31.3 | 8.00 (5.35-11.95) | 0.74 | 0.93 (0.61-1.42) | 0.74 |
| Respiratory rate (breaths/min) ≤ 8 or ≥ 30 | No (ref.) | 77 (44.3) | 109.5 | 6.76 (5.37-8.50) |  |  |  |
|  | Yes | 112 (60.5) | 93.9 | 11.71 (9.71-14.14) | 0.0035 | 1.53 (1.15-2.05) | 0.0040 |
| Heart rate (bpm), ≥130 OR ≥ 1503 | No (ref.) | 179 (53.4) | 185.4 | 9.39 (8.09-10.90) |  |  |  |
|  | Yes | 24 (36.4) | 47.6 | 5.04 (3.35-7.58) | 0.0086 | 0.57 (0.37-0.87) | 0.010 |
| RLS > 3 | No (ref.) | 164 (48.7) | 205.7 | 7.78 (6.66-9.09) |  |  |  |
|  | Yes | 40 (61.5) | 28.2 | 13.83 (10.04-19.04) | 0.0044 | 1.64 (1.16-2.32) | 0.0052 |
| Ongoing seizures | No (ref.) | 200 (51.7) | 222.2 | 8.78 (7.62-10.10) |  |  |  |
|  | Yes | 4 (26.7) | 11.7 | 3.41 (1.17-9.96) | 0.080 | 0.43 (0.16-1.15) | 0.091 |
| Signs of infection | No (ref.) | 135 (51.7) | 147.4 | 8.82 (7.42-10.48) |  |  |  |
|  | Yes | 69 (48.9) | 86.5 | 7.97 (6.29-10.12) | 0.36 | 0.87 (0.65-1.17) | 0.37 |
| Oxygen saturation <90%  2 Systolic blood pressure <90 mmHg  3 Regular ≥130 or irregular ≥ 150  *Only one patient had leukemia  Analyses reported both with CFS treated as a continuous and a categorical variable, respectively.  CFS, Clinical frailty scale; CCI, Charlson Comorbidity Index; MI, Myocardial Infarction; CHF, Congestive Heart Failure; PAD, Peripheral Arterial Disease; CVD, Cerebrovascular disease; COPD, Chronic Obstructive Pulmonary Disease; CKD, Chronic Kidney Disease; BPM, beats per minute; RLS, Reaction Level Scale; BPM, beats per minute | | | | | | | |
