# Supplementary figures and images for: Clinical Frailty Scale score is a predictor of short-, mid- and long-term mortality in critically ill older adults (≥ 70 years) admitted to the emergency department: an observational study
Source: BMC Geriatr. 2024 Oct 21;24:852. doi: 10.1186/s12877-024-05463-7 (PMC11492669; doi:10.1186/s12877-024-05463-7)

**Additional file 3.** Kaplan-Meier (KM) survival curves (one year) versus CFS-levels


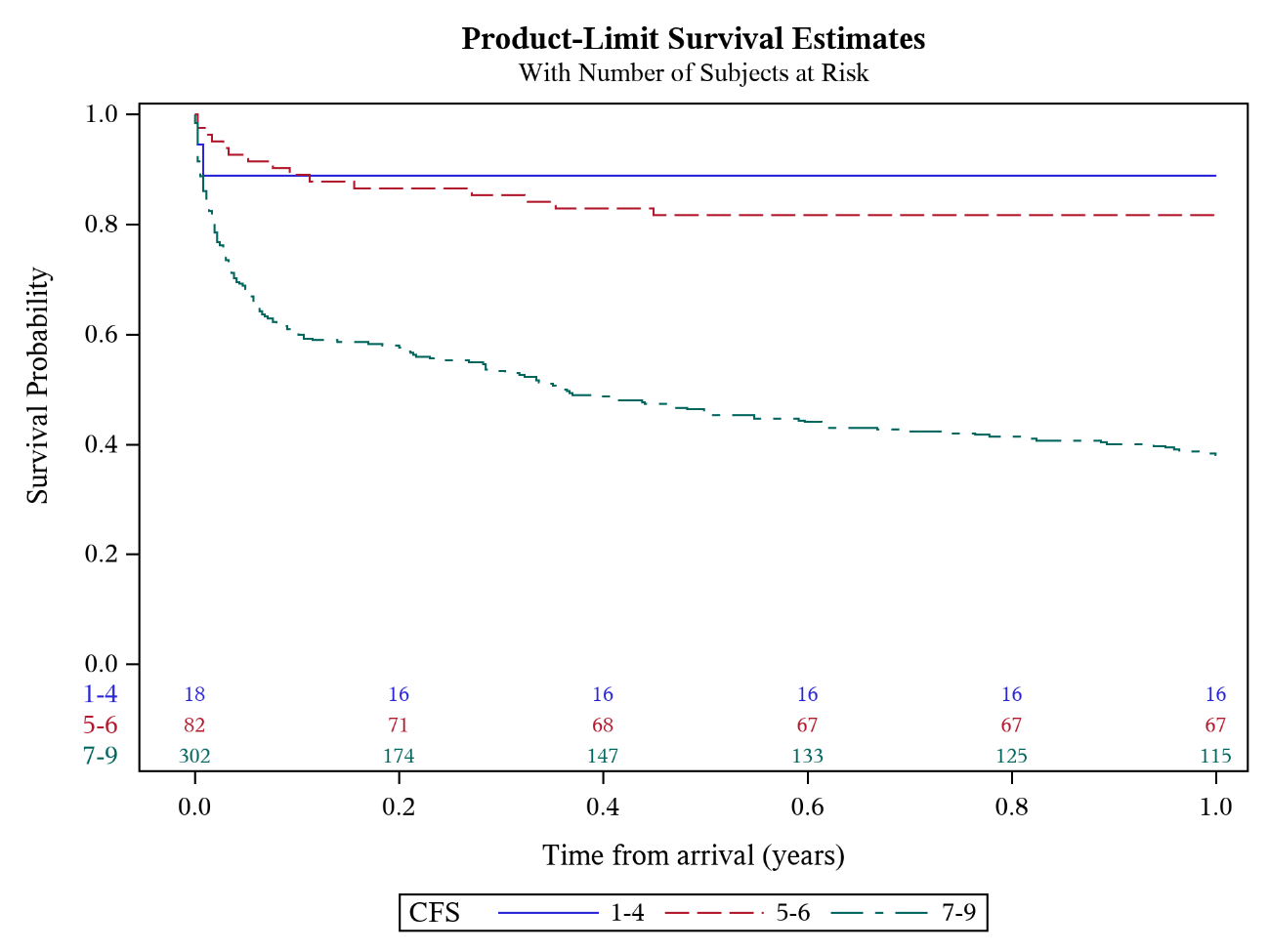


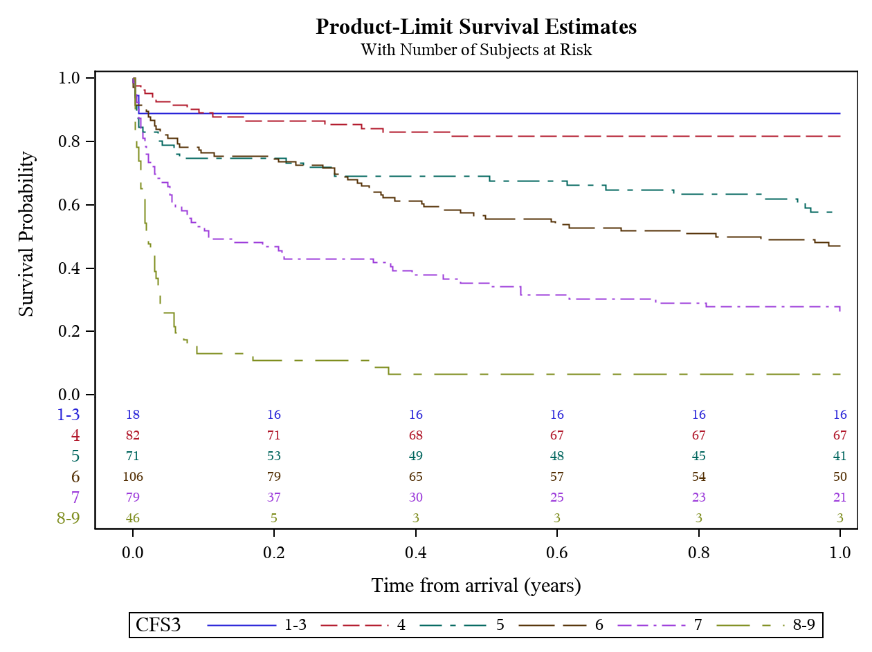

Supplement: Supplementary file 3 — Additional file 3 Kaplan-Meier (KM) survival curves (one year) versus CFS-levels [file 12877_2024_5463_MOESM3_ESM.docx]
