# Supplementary material for: Clinical Frailty Scale score is a predictor of short-, mid- and long-term mortality in critically ill older adults (≥ 70 years) admitted to the emergency department: an observational study
Source: BMC Geriatr. 2024 Oct 21;24:852. doi: 10.1186/s12877-024-05463-7 (PMC11492669; doi:10.1186/s12877-024-05463-7)
Supplement: Supplementary file 4 — Additional file 4 Unadjusted analysis regarding all-cause mortality 6.5-7.5 years after discharge [file 12877_2024_5463_MOESM4_ESM.docx]

| **Additional file 4.** Unadjusted analysis regarding all-cause mortality 6.5-7.5 years after discharge | | | | | | | |
| --- | --- | --- | --- | --- | --- | --- | --- |
| **Variable** | **Value** | **n (%) of event** | **Total time (Years)** | **Events per 10 Years (95% CI)** | **Log-Rank p-value** | **HR (95% CI)** | **Cox p-value** |
| Age | 69.7-79.1 | 64 (64.0) | 420.5 | 1.52 (1.19-1.95) |  |  |  |
|  | 79.4-85.4 | 92 (91.1) | 274.5 | 3.32 (2.69-4.08) |  |  |  |
|  | 85.6-98.3 | 97 (96.0) | 174.9 | 5.55 (4.53-6.78) | <.0001 | 1.07 (1.05-1.09) | <.0001 |
| Sex | Female (ref.) | 116 (84.1) | 382.0 | 3.04 (2.53-3.65) |  |  |  |
|  | Male | 137 (83.5) | 487.9 | 2.79 (2.35-3.30) | 0.61 | 0.94 (0.73-1.20) | 0.61 |
| CFS-score | 1-4 | 52 (57.1) | 468.2 | 1.11 (0.84-1.46) |  |  |  |
|  | 5-6 | 132 (93.0) | 327.0 | 4.01 (3.37-4.76) |  |  |  |
|  | 7-9 | 69 (100.0) | 74.6 | 9.24 (7.27-11.75) | <.0001 | 1.80 (1.62-1.99) | <.0001 |
| CFS-score (categorical)  5 versus 1-4 2.69 (1.81-4.01) <.0001  6 versus 1-4 4.37 (3.04-6.27) <.0001  7 versus 1-4 6.39 (4.28-9.52) <.0001  8 versus 1-4 20.18 (11.07-36.8) <.0001  **CCI-variables** | | | | | | | |
| CCI-score | 0-1 | 101 (72.7) | 516.2 | 1.94 (1.59-2.36) |  |  |  |
|  | 2-2 | 64 (92.8) | 176.5 | 3.63 (2.82-4.65) |  |  |  |
|  | 3-9 | 88 (93.6) | 177.1 | 4.97 (4.02-6.14) | <.0001 | 1.25 (1.17-1.34) | <.0001 |
| Previous MI | No (ref.) | 199 (81.9) | 723.8 | 2.74 (2.38-3.15) |  |  |  |
|  | Yes | 53 (91.4) | 144.8 | 3.66 (2.78-4.82) | 0.14 | 1.26 (0.93-1.70) | 0.14 |
| CHF | No (ref.) | 185 (79.7) | 752.8 | 2.44 (2.11-2.83) |  |  |  |
|  | Yes | 68 (97.1) | 117.0 | 5.81 (4.56-7.40) | <.0001 | 2.01 (1.51-2.67) | <.0001 |
| PAD | No (ref.) | 236 (83.4) | 811.6 | 2.90 (2.55-3.29) |  |  |  |
|  | Yes | 17 (89.5) | 58.2 | 2.92 (1.75-4.86) | 0.99 | 1.00 (0.61-1.63) | 0.99 |
| CVD | No (ref.) | 191 (82.3) | 717.3 | 2.66 (2.31-3.07) |  |  |  |
|  | Yes | 62 (88.6) | 152.6 | 4.00 (3.10-5.16) | 0.016 | 1.42 (1.07-1.90) | 0.017 |
| Dementia | No (ref.) | 203 (80.6) | 790.4 | 2.56 (2.22-2.94) |  |  |  |
|  | Yes | 50 (100.0) | 79.4 | 6.29 (4.74-8.36) | <.0001 | 2.18 (1.59-2.98) | <.0001 |
| COPD | No (ref.) | 189 (82.2) | 679.9 | 2.77 (2.39-3.19) |  |  |  |
|  | Yes | 64 (88.9) | 190.0 | 3.37 (2.63-4.32) | 0.30 | 1.16 (0.87-1.54) | 0.30 |
| Diabetes | No (ref.) | 191 (82.7) | 675.5 | 2.81 (2.44-3.25) |  |  |  |
|  | Without Complications | 48 (87.3) | 151.5 | 3.17 (2.37-4.23) |  | 1.09 (0.79-1.49) | 0.60 |
|  | With Complications | 14 (87.5) | 42.9 | 3.27 (1.85-5.77) | 0.58 | 1.09 (0.63-1.88) | 0.75 |
| Moderate to severe CKD | No (ref.) | 227 (83.2) | 817.5 | 2.76 (2.43-3.15) |  |  |  |
|  | Yes | 26 (89.7) | 52.4 | 4.96 (3.32-7.42) | 0.022 | 1.60 (1.07-2.41) | 0.023 |
| Tumor | No (ref.) | 230 (83.0) | 822.0 | 2.79 (2.45-3.17) |  |  |  |
|  | Without Metastases | 16 (88.9) | 41.3 | 3.87 (2.28-6.56) |  | 1.31 (0.79-2.18) | 0.29 |
|  | Metastatic | 7 (100.0) | 6.6 | 10.66 (4.23-26.88) | 0.78 | 2.87 (1.35-6.11) | 0.0063 |
| Lymphoma | No (ref.) | 248 (83.5) | 857.8 | 2.88 (2.54-3.26) |  |  |  |
|  | Yes | 5 (100.0) | 12.1 | 4.14 (1.20-14.32) | 0.45 | 1.41 (0.58-3.41) | 0.45 |
| **Vital signs on admissions** | | | | | | | |
| Obstructive airway | No (ref.) | 238 (83.5) | 822.4 | 2.88 (2.54-3.28) |  |  |  |
|  | Yes | 15 (88.2) | 47.5 | 3.16 (1.83-5.46) | 0.85 | 1.05 (0.62-1.78) | 0.85 |
| Hypoxia1 | No (ref.) | 110 (76.4) | 542.5 | 2.01 (1.66-2.43) |  |  |  |
|  | Yes | 139 (90.8) | 309.4 | 4.49 (3.80-5.31) | <.0001 | 1.99 (1.54-2.56) | <.0001 |
| Hypotension2 | No (ref.) | 218 (83.5) | 758.3 | 2.86 (2.50-3.27) |  |  |  |
|  | Yes | 33 (84.6) | 109.3 | 3.02 (2.12-4.30) | 0.87 | 1.03 (0.72-1.49) | 0.87 |
| Respiratory rate (breaths/min) ≤ 8 or ≥ 30 | No (ref.) | 106 (76.8) | 451.4 | 2.33 (1.92-2.82) |  |  |  |
|  | Yes | 118 (91.5) | 308.6 | 3.82 (3.19-4.59) | 0.0012 | 1.54 (1.18-2.01) | 0.0013 |
| Heart rate (bpm), ≥130 OR ≥ 1503 | No (ref.) | 211 (86.5) | 645.7 | 3.25 (2.84-3.73) |  |  |  |
|  | Yes | 41 (71.9) | 223.2 | 1.84 (1.34-2.51) | 0.0025 | 0.60 (0.43-0.84) | 0.0028 |
| RLS >3 | No (ref.) | 221 (83.7) | 768.2 | 2.86 (2.51-3.27) |  |  |  |
|  | Yes | 32 (84.2) | 101.7 | 3.15 (2.20-4.50) | 0.67 | 1.08 (0.75-1.57) | 0.67 |
| Ongoing seizures | No (ref.) | 242 (83.7) | 837.5 | 2.88 (2.54-3.27) |  |  |  |
|  | Yes | 11 (84.6) | 32.4 | 3.40 (1.76-6.55) | 0.76 | 1.10 (0.60-2.01) | 0.76 |
| Signs of infection | No (ref.) | 159 (83.2) | 534.6 | 2.96 (2.53-3.46) |  |  |  |
|  | Yes | 94 (84.7) | 335.3 | 2.80 (2.29-3.44) | 0.71 | 0.95 (0.74-1.23) | 0.71 |
| 1 Oxygen saturation <90%  2 Systolic blood pressure <90 mmHg  3 Regular ≥130 or irregular ≥ 150  CFS, Clinical frailty scale; CCI, Charlson Comorbidity Index; MI, Myocardial Infarction; CHF, Congestive Heart Failure; PAD, Peripheral Arterial Disease; CVD, Cerebrovascular disease; COPD, Chronic Obstructive Pulmonary Disease; CKD, Chronic Kidney Disease; BPM, beats per minute; RLS, Reaction Level Scale;  Analyses reported both with CFS treated as a continuous and a categorical variable, respectively. | | | | | | | |
